# Cuproptosis‐related gene DLAT is a biomarker of the prognosis and immune microenvironment of gastric cancer and affects the invasion and migration of cells

**DOI:** 10.1002/cam4.70012

**Published:** 2024-07-19

**Authors:** Yanyu Peng, Ruimeng Shi, Siwen Yang, Jiayi Zhu

**Affiliations:** ^1^ Department of Histology and Embryology Shenyang Medical College Shenyang Liaoning China; ^2^ Shenyang Medical College Shenyang Liaoning China

**Keywords:** cuproptosis, DLAT, gastric cancer, immune infiltration, prognosis

## Abstract

**Objective:**

Cuproptosis is a novel cell death dependent on mitochondrial respiration and regulated by copper. This study aimed to investigate the cuproptosis‐related gene DLAT potential value in gastric cancer (GC).

**Methods:**

Bioinformatics was used to analyze DLAT expression. DLAT expression in GC cell lines was detected using qRT‐PCR. Cell proliferation ability was assessed using CCK8 and cell cycle assay. Cell migration and invasion were assessed using wound healing and transwell assay. A prognostic assessment was performed through survival and Cox regression analysis. DLAT protein expression was analyzed through HPA immunohistochemistry. Biological functions and processes were analyzed through GO and KEGG enrichment analysis and PPI. Correlation with immune cell infiltration and immune checkpoint genes was analyzed for DLAT.

**Results:**

DLAT expression was upregulated in GC tissues and cells and correlated with shorter survival for patients. Age, gender, histological typing, lymph node metastasis, and distant metastasis were identified as independent prognostic factors affecting OS in GC. DLAT protein was upregulated in GC. The biological functions and pathways enriched in DLAT were mainly linked to mitochondrial respiration and the TCA cycle. The expression of DLAT was found to be positively correlated with the infiltration of Th and Th2 immune cells and only positively correlated with the expression of the BTN2A1 immune checkpoint gene.

**Conclusion:**

DLAT has the potential to serve as a prognostic assessment factor in GC. The expression of DLAT was correlated with immune infiltration and tumor immune escape, providing a new target for immunotherapy of GC.

## Graphical abstract

Cuproptosis‐related DLAT may play an important role in the prognostic assessment of gastric cancer. Taking advantage of the energy metabolism pathway of cuproptosis could hold promise for targeting tumor cell death through combination therapy. DLAT may be associated with tumor immune cell infiltration and involved in tumor immune escape by interfering with immune checkpoints.

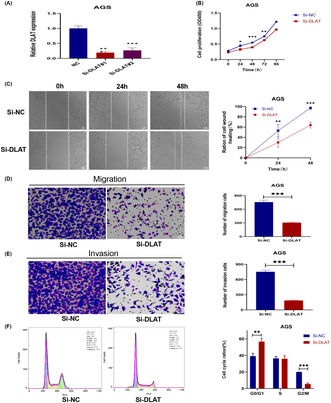

## 1 INTRODUCTION

Although there have been significant advancements in the early diagnosis and targeted treatments of gastric cancer (GC) in recent years, the prevention and treatment of GC remain a pressing global public health issue.^1^, ^2^ The China Guidelines for Gastric Cancer Screening and Early Diagnosis and Treatment (2022, Beijing)^3^ emphasize that screening, early diagnosis, and treatment among high‐risk individuals can effectively reduce morbidity and mortality. Geographical differences also play a role in the incidence of GC worldwide.^4^ However, due to its hidden onset and atypical symptoms, early diagnosis and treatment are crucial for improving survival rates.^5^

Copper is a vital trace element in the human body and is involved in several physiological processes, such as iron transport, mitochondrial respiration, and detoxification of oxygen‐free radicals.^6^ The body maintains intracellular copper levels within physiological ranges through active homeostatic mechanisms using trans‐concentration gradients to prevent free copper overload, which can damage cells. In 2022, Peter et al.^7^ presented cuproptosis as a copper‐dependent cell death dependent on mitochondrial respiration. They identified seven genes (FDX1 and six genes involved in proteolipoylation LIAS, LIPT1, DLD, DLAT, PDHA1, and PDHB) as the key genes promoting cellular cuproptosis. The primary target of cuproptosis‐induced cell death was mitochondrial dysfunction. In cells where oxidative phosphorylation was the main energy metabolism, the FDX1/LIAS‐mediated lipoic acid pathway induced excess copper ions bound to dihydrolipoamide S‐acetyltransferase (DLAT) to form oligomerized DLAT proteins, which affected the tricarboxylic acid (TCA) cycle, triggered proteotoxic stress, and induced cell death. Therefore, mitochondria were the main target for inducing cellular cuproptosis, and cellular aerobic respiratory energy metabolism was necessary for cuproptosis.^8^ In addition, these genes also served as key regulators of copper–carrier‐induced cell death. Changes in cellular copper content affected the onset and progression of tumors.^9^ The role of DLAT in cancer may be involved in metabolic reprogramming and affects tumor growth and survival.^10^, ^11^ Furthermore, it has been reported that copper carriers may have an anticancer effect in breast cancer^12^ and melanocytoma^13^ by inducing cellular copper death in tumor cells with excess copper. These carriers utilized the abundance of copper in tumor tissues to increase the susceptibility of tumor cells to oxidative stress. Additionally, the cuproptosis properties of these carriers may aid in predicting the prognosis and immune microenvironment (IME) of patients with breast cancer.^14^

The tumor microenvironment (TME) of GC involves complex interactions between cancer cells and immune cells. Immune cells infiltrate tumors and can either support or inhibit tumor progression. The composition of immune cells (such as T cells, macrophages, and natural killer cells) and their activation status can be influenced by metabolic changes within the TME.^15^ The changes in metabolite levels (such as lactate and ketones) could modulate immune cell behavior either promoting an immunosuppressive environment favorable to cancer or stimulating an immune response against the tumor.^16^ Altered energy metabolism, particularly glycolysis as the dominant energy metabolism, was one of the hallmarks of GC cells. As part of the pyruvate dehydrogenase (PDC) complex, DLAT catalyzes critical reactions in cellular metabolism by converting pyruvate to acetyl coenzyme A (acetyl‐CoA), linking glycolysis and the TCA cycle. The role of DLAT in cancer may be involved in metabolic reprogramming and affects tumor growth and survival.^10^, ^11^ The copper content was significantly increased in tumor tissues compared to normal tissues, which affected the biological behavior of tumors.^17^, ^18^, ^19^ The cuproenzyme LOX was involved in the invasion and migration of tumor cells,^20^ and the copper sulfate (CuSO4) was demonstrated to promote the growth of breast cancer in rats.^21^ Therefore, cuproptosis‐related genes may provide and guide the development of new treatment programs.^22^ However, the role of DLAT in GC is unknown, and it is uncertain whether it has the potential to broaden therapeutic strategies for tumors by relying on its importance in cuproptosis. In this study, we investigated the expression of the cuproptosis‐related gene DLAT in GC and its value in assessing survival, which may provide new ideas for the treatment and prognostic assessment of GC. Furthermore, this study observed the effects of DLAT on the biological behavior of GC cells, as well as analyzed the relationship between DLAT and immune cell infiltration and immune checkpoint genes. The role of DLAT in TME was also explored in depth. The relationship between the induction of tumor cell death, copper, DLAT, and cuproptosis was also discussed deeply, with the potential to provide new ideas and identify new therapeutic targets for future specific therapies.

## 2 METHODS

### 2.1 Data and processing methods

RNAseq data was downloaded from the TCGA for the STAR (https://portal.gdc.cancer.gov/) process of the TCGA‐STAD project and extracted in TPM format, including 32 cases of paracancer and 375 cases of GC tissues. The data were processed using the log2 (value+1) method. Normal tissue sequencing data from the UCSC XENA database (https://xenabrowser.net/datapages/) of 174 GTEx cases was uniformly processed using the Toil process.^23^ The TNMplot (www.tnmplot.com) is a tool for comparing gene expression in normal, tumor, and metastatic tissues across various databases, including TCGA, GEO, GTEx, and TARGET. The data set included 56,090 unique samples 15,648 normal and 40,442 tumor samples, respectively, from the TCGA, GEO, GTEx, and TARGET databases.^24^

### 2.2 Gene expression and prognostic correlation analysis

The analysis of differential expression of DLAT among various tissue samples was conducted using R software (version 4.2.1) and Deseq2^25^ and stats (version 4.2.1)and car packages. Visualization was performed using the ggplot2 package (version 3.3.3).^26^ The GC node was selected in the KM plotter (www.kmplot.com), and DLAT expression was grouped according to the automatically selected best cutoff value, and survival curves for OS (overall survival) and PFS (progression‐free survival) were plotted.^27^ Area under the curve (AUC) receiver operating characteristic (ROC) analysis was performed on the data using the pROC package. Univariate Cox analysis with R software was used to assess risk ratios (HR) and *p*‐values for clinical factors. Clinical factors with *p* < 0.1 were selected for multivariate Cox regression analysis. Prognostic OS nomographs were constructed using the rms package (version 6.2‐0) and the survival package (version 3.2‐10).^28^ The constructs' parameters were set as follows: a sample size of 40 cases per group of repeated calculations, a frequency of 200 repeated calculations, and the method was chosen as boot. The plots were visualized using the ggplot2 package.

### 2.3 Analysis of protein expression and immunohistochemistry

The protein expression data of DLAT from TCGA‐STAD provided by UALCAN (https://ualcan.path.uab.edu/) were utilized for quantitative analysis.^29^, ^30^ The qualitative protein expression of DLAT (antibody number CAB003782) was analyzed by GC and normal gastric mucosa tissues in the Human Protein Atlas (HPA) online database (https://www.proteinatlas.org/). The HPA was established to create a comprehensive map of the human proteome through the integration of various omics technologies. This enables the exploration of tissue‐specific proteomes in various tissues and organs and the analysis of tissue profiles for specific protein classes.^31^ As stated in the HPA introduction, the antibody was employed to generate tissue‐based immunohistochemistry images, which were subsequently annotated (shown on the website) by pathologists for all sampled tissues. All data can be downloaded and cross‐referenced upon request.

### 2.4 Cell lines and cell culture

The following GC cell lines were used in the experiments: HGC‐27, MKN‐45, MGC‐803, AGS, and SNU‐1, along with human gastric mucosal epithelial cells GES‐1 (Servicebio, Immocell, China). The cell lines confirmed using short tandem repeat analysis were utilized in this study. They were cultured in RPMI 1640 medium (Gibco, USA) supplemented with 10% fetal bovine serum (FBS, Clark Bioscience), 100 U/mL penicillin, and 100 μg/mL streptomycin. The cultures were maintained at 37°C with 5% CO_2_.

### 2.5 Total RNA extraction and real‐time quantitative PCR (qRT‐PCR)

RNA was extracted using the RNA Easy Fast Tissue/Cell Kit (Tiangen, China) following the provided protocol. Reverse transcription was performed using the primeScript RT Master Mix Kit (Takara, China). The relative expression of mRNA was detected using the SYBR Green Master Mix Kit (Takara, China). The primer sequences are the following:

DLAT F sequence (5′‐3′): ACTCCCCAGCCTTTAGCTC;

DLAT R sequence (5′‐3′): CAATCCCTTTCTCTACTGCCAAC.

GAPDH F sequence (5′‐3′): GAGTCAACGGGATTTGGTCGT;

GAPDH R sequence (5′‐3′): TTGATTTTGGAGGGATCTCG.

The tested genes were normalized with GAPDH. Each sample assay was run in 7500Fast (ABI, USA) and repeated three times.

### 2.6 SiRNA transfection

Target‐specific siRNA (si‐DLAT) and negative control (si‐NC) were provided by GenePharma (China) and transfected into AGS cells cultured in six‐well plates. The AGS cells were transfected with 20 nM siRNA using Lipo 6000 (Beyotime, China) transfection reagent. The silencing efficiency was assessed by qRT‐PCR after 48 h.

### 2.7 CCK8 proliferation assay

The CCK8 kit (MCE, USA) was used to detect cell proliferation. 2 × 10^3^/well cells were seeded in 96‐well plates with 100 μL and incubated at 37°C with 5% CO_2_. At 0, 24, 48, 72, and 96 h, the CCK8 solution was diluted 1:10 with RPMI‐1640 medium containing 10% FBS, and 100 μL of the dilution solution was added to each well to be detected. The cells were incubated for 2 h. The OD value at 450 nM was read using the enzyme‐labeled instrument (Molecular Devices, USA). The assay was in triplicate and repeated at least three times.

### 2.8 Wound healing assay

The wound healing assay was performed to assess the ability of the cells to migrate. Cells were cultured at 13 × 10^4^/well in 12‐well plates. After 90% cell fusion, the cell‐free area was scratched with a 200 μL pipette tip, washed 2–3 times with PBS, and the cells were incubated in 5% low‐serum medium, and the scratches were photographed under a microscope at 0, 24, and 48 h. The assay was in triplicate and repeated at least three times.

### 2.9 Transwell assay

The Transwell migration assay was performed with cells in the logarithmic growth phase. When the cell concentration was about 3 × 10^5^/well in serum‐free medium, 200 μL of cell suspension was added to the upper chamber of the transwell, and 500 μL of RPMl‐1640 medium containing 20% FBS was added to the lower chamber, and the cells were incubated for 48 h at 37°C with 5% CO_2_. For the Transwell invasion assay, the Matrigel gel should be diluted with serum‐free medium: Matrigel gel = 7:1, and 50 μL of the dilution was added to the upper chamber of the transwell and incubated at 37°C for 1 h. After resuspending the cells into single cell suspension with the serum‐free medium at a concentration of about 4 × 10^5^/well, 200 μL of cell suspension was added to the upper chamber of the transwell, and 500 μL of RPMI‐1640 medium containing 20% FBS was added to the lower chamber. The cells were incubated at 37°C under 5% CO_2_ for 48 h. After washing with PBS, the cells on the surface of the chamber were fixed with 4% paraformaldehyde for 15 min, then stained with 0.1% crystal violet for 20 min, and then photographed with an inverted microscope and counted the migrated cells, and the average of cell counts of the three fields of view was recorded for analysis. The assay was in triplicate and repeated at least three times.

### 2.10 Flow cytometry

The cell cycle was detected by flow cytometry. Cultured experimental cells were stained with propidium iodide (PI, Beyotime, China) for cell cycle detection. Cells from each group of logarithmic growth phase (about 6 × 10^6^/well) were collected in EP tubes, washed three times with PBS, precooled, fixed in 70% alcohol, and kept at 4°C overnight. The treated cells were stained with PI for 30 min at 37°C. Flow cytometry analysis was performed using a FACSCanto II flow cytometer (FACSCalibur BD, USA). Experiments were repeated three times and data were processed using Flow Jo software (Verity Software House, Topsham, ME).

### 2.11 Analysis of protein–protein interactions (PPI)

The STRING database was used to analyze proteins that interacted with the DLAT protein.^32^ When searching for reciprocal proteins of DLAT, the species was limited to homo sapiens, and the parameter setting of the minimum required interaction score item confidence was selected: 0.3. The GEPIA2 website was searched for DLAT similar genes, the cancer species was selected as STAD, and the quantity was limited to 100. Using the plug‐in cytoHubba of Cytoscape (version 3.8.0),^33^ the top 15 hub genes were selected at Hubba nodes by the degree calculation method to get the final PPI network graph. The Gene Ontology (GO) and Kyoto Encyclopedia of Genes and Genomes (KEGG) pathway enrichment analyses were undertaken by the clusterProfiler package [3.14.3 version]. The conditions of *P*.adj < 0.05 and *q* value < 0.2 were met and visualized by the ggplot2 package.

### 2.12 Immune cell infiltration and immune checkpoint co‐expression analysis

The immune infiltration analysis was performed using ssGSEA (single sample gene set enrichment analysis) (built‐in algorithm in GSVA package [version 1.34.0]),^34^ and markers for 24 immune cells were obtained from published literature.^35^ The median value of DLAT expression in GC was divided into low and high expression groups, and co‐expression correlation heatmaps were plotted with 79 immune checkpoint genes,^36^ which were visualized with the ggplot2 package.

### 2.13 Statistical analysis

Parametric comparisons were performed using *t*‐tests, and data were statistically analyzed by mean ± standard deviation (SD). Nonparametric tests for two independent samples were performed with the Mann–Whitney *U* test. The Kaplan–Meier analysis for survival, the log‐rank test for comparing survival between groups, univariate and multivariate Cox regression analyses for evaluating independent risk factors, and correlation analyses were performed using Spearman's method. All statistical analyses were performed by GraphPad Prism 7.0, SPSS (version 22.0), or R software (version 4.1.2). *p* < 0.05 was the threshold of significance. The statistical significance was described as follows ns, not significant; * *p* < 0.05; ** *p* < 0.01; *** *p* < 0.001.

## 3 RESULTS

### 3.1 DLAT expression is dysregulated in Pan‐cancer and upregulated in gastric cancer

**FIGURE 1:**
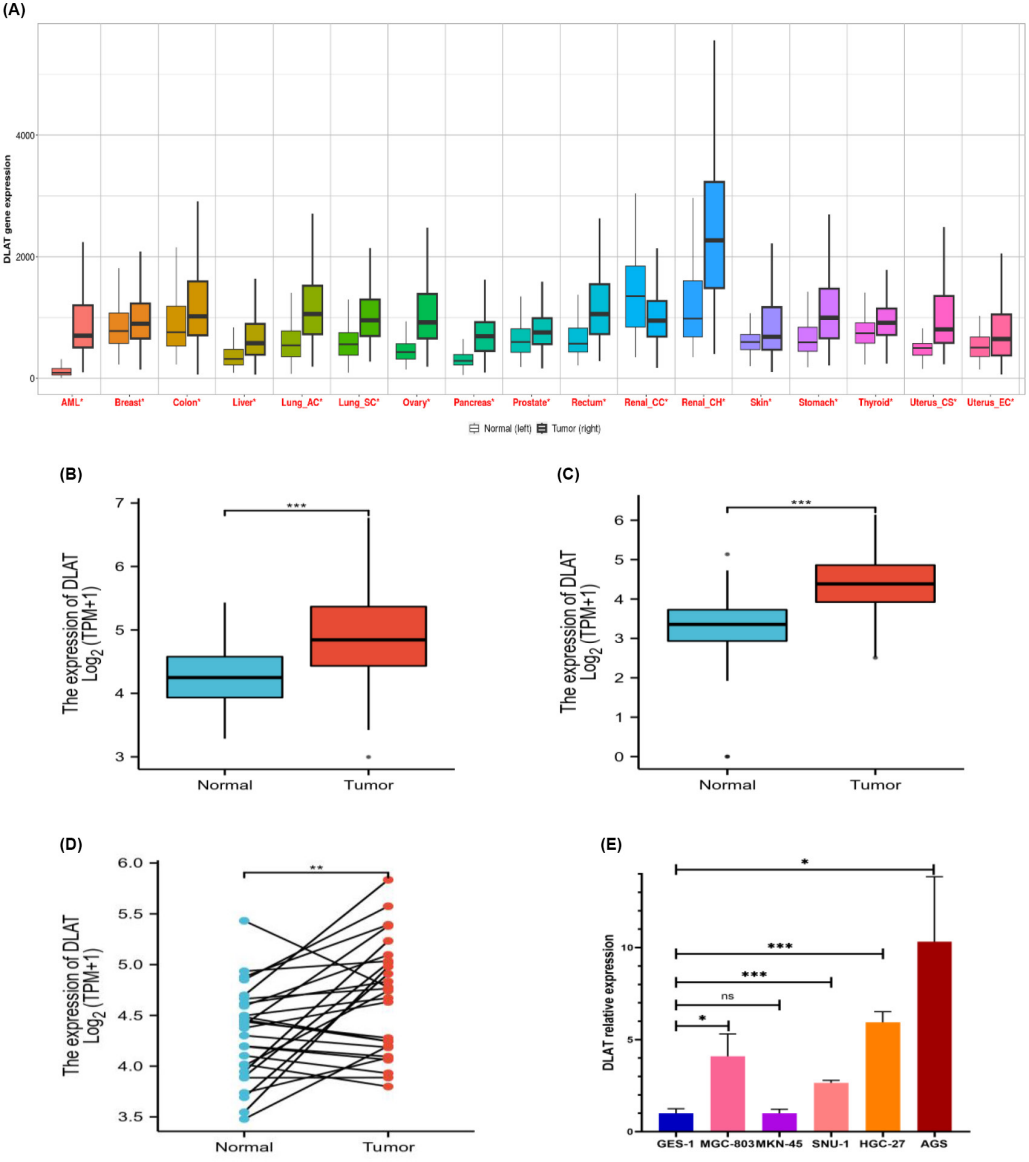
DLAT expression in gastric cancer tissues and cells (A). DLAT expression in Pan‐cancer (B). DLAT expression was upregulated in the unpaired GC group in TCGA‐STAD compared to the normal group (C). DLAT expression was upregulated in the GC group in TCGA‐STAD in combination with GTEx normal samples compared to the normal group (D). DLAT expression was upregulated in the paired GC group in TCGA‐STAD (E). DLAT expression was upregulated in MGC‐803, SNU‐1, HGC‐27, and AGS compared to GES‐1. * *p* < 0.05, ** *p* < 0.01, *** *p* < 0.001.

The pan‐cancer analysis displayed the expression range for the DLAT gene across all tissues in all available normal and tumor RNA‐Seq data. The pan‐cancer analysis tool also compared normal and tumor samples across 22 tissue types simultaneously, but only 17 tumor types showed significant differences (Figure 1A). For expression analysis, firstly, unpaired samples of TCGA‐STAD were selected. The results indicate that the average expression of DLAT at the tissue level was higher in the tumor group than in the normal group, and the difference between the two groups was statistically significant (*p* < 0.001; Figure 1B). Additionally, the analysis combined TCGA‐STAD RNA‐seq data with GTEx normal samples. The results showed a statistically significant difference (*p* < 0.001) in expression levels between the tumor and normal groups, with the tumor group exhibiting significantly higher expression levels (Figure 1C). The analysis of paired TCGA‐STAD samples revealed that the expression levels in the tumor group were significantly higher than those in the Normal group, and the difference between the two groups was statistically significant (*p* = 0.003; Figure 1D). DLAT expression was detected in GC cell lines. The results showed significant upregulation of DLAT expression in MGC‐803, SNU‐1, HGC‐27, and AGS cell lines compared to the GES‐1 (Figure 1E). Taken together, these results demonstrated that DLAT expression was dysregulated in tumors and significantly upregulated in GC tissues and cells.

### 3.2 Association of DLAT expression with clinicopathologic parameters

**FIGURE 2:**
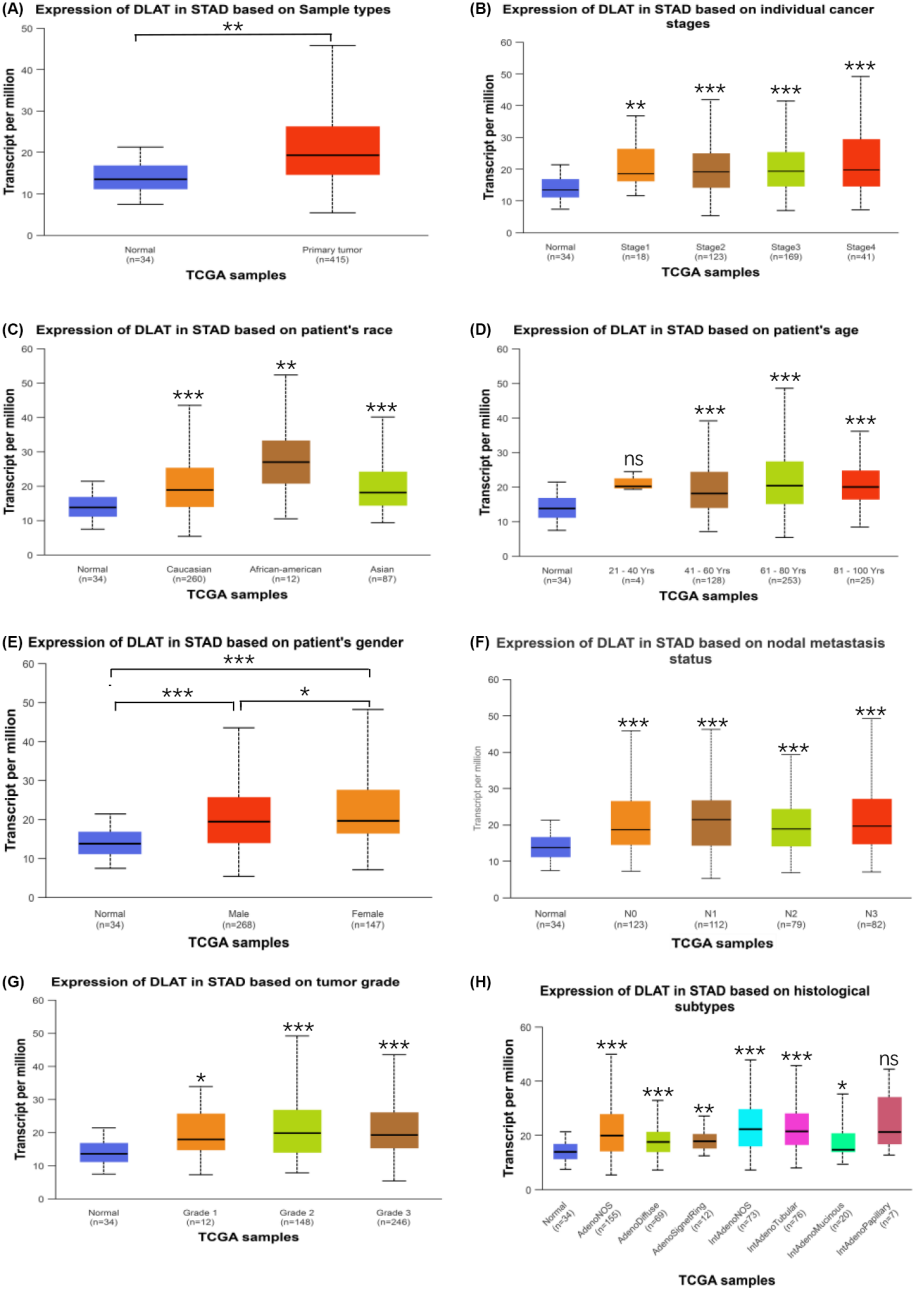
Association of DLAT expression with clinicopathologic parameters (A). The expression of DLAT in the Normal group and tumor group was analyzed by TCGA (B) Individual cancer stages (C). Race (D), Age (E), Gender (F), N (G), Grade (H). Histological subtypes. *p < 0.05, **p < 0.01, ***p < 0.001.

The relationship between DLAT expression and clinicopathological features of GC patients in TCGA data was analyzed. The results showed that DLAT expression was upregulated in primary tumors compared to the normal group (Figure 2A), and there were significant differences in DLAT expression among different pathological factors, such as pathological stage, race, age, gender, N stage, histological grade, and histological subtype (Figure 2B–H). That is, the higher the subgroups of males over 40 years of age, African Americans, pathological stage, histological grade, and number of lymph node metastases, the higher the DLAT expression. However, there were no significant differences in age less than 40 years and histologic subtype of the Int Adeno Papillary subgroup compared to the normal group.

### 3.3 The high expression of DLAT in gastric cancer is associated with poorer prognosis

**FIGURE 3:**
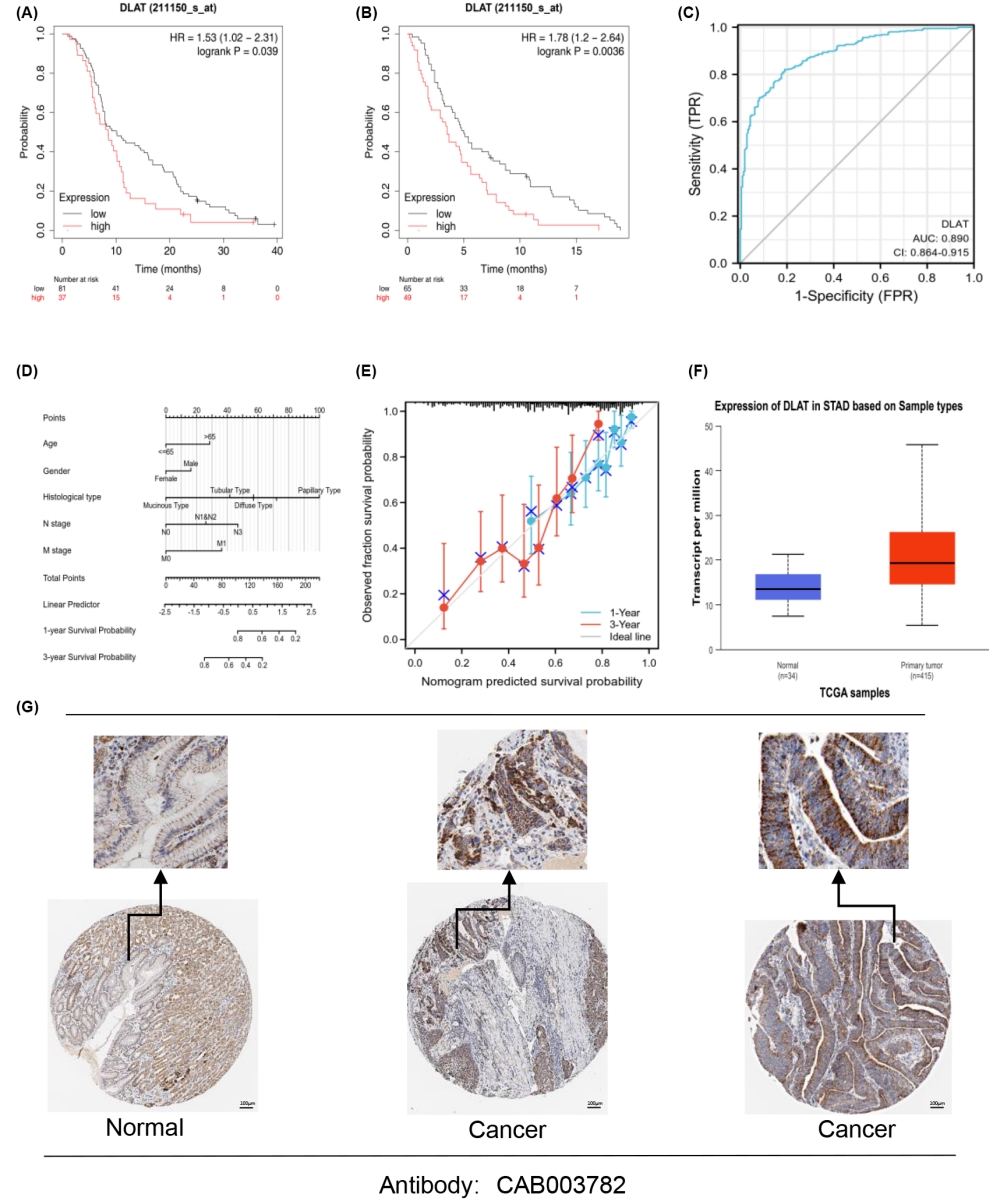
Prognostic analysis and protein expression analysis of DLAT in gastric cancer (A). Kaplan–Meier prognosis analysis of OS and PFS of GC patients (B). Kaplan–Meier prognosis analysis of PFS of GC patients (C). ROC curve. Nomograms (D) and calibration (E) curves predicting 1‐and 3‐year OS of GC patients (F). DLAT protein expression analysis (G). The expression of DLAT protein in GC was analyzed by immunohistochemistry. *p < 0.05, **p < 0.01, ***p < 0.001.

To assess the relationship between DLAT expression and the prognosis of GC with OS (Figure 3A) and PPS (Figure 3B). The KM Plotter displayed that patients with high DLAT expression had significantly shorter survival and poorer prognoses than those with low expression. The ROC curve was used to analyze the TCGA‐STAD data to evaluate the diagnostic value of DLAT (Figure 3C). The study found that DLAT could help the diagnosis of GC. The above results indicated that the high expression of DLAT in GC was significantly correlated with the poor prognosis of the patients. In addition, DLAT had a higher diagnostic value for GC.

### 3.4 Univariate/Multivariate Cox regression analysis and prognostic analysis

The regression analysis was used to evaluate the predictive value of DLAT expression and clinicopathologic features on the prognosis of GC patients. Univariate and multivariate Cox risk regression models of OS in GC patients were constructed, and the results are presented in Table 1. The multivariate regression analyses included variables that were significant in univariate Cox analyses. The results showed that age (>65 years old), female, histologic type, lymph node metastasis (N3), and distant metastasis (M1) were the independent prognostic predictive factors affecting the OS of patients with GC (*p* < 0.05). The consistency C‐index was 0.691 (95% CI 0.667–0.716). Additionally, a nomogram plot was constructed (Figure 3D) by identifying variables from the Cox regression to confirm that DLAT was a good predictor of patient prognosis at 1 and 3 years. The calibration curves (Figure 3E) also verified that the 1‐ and 3‐year trends were close to the ideal line, and the survival probability of more samples was concentrated between 0.6 and 0.8. These findings demonstrated that DLAT expression has a value as an independent predictor of prognosis in GC patients.

**TABLE 1:** Univariate and multivariate cox regression analyses of DLAT.

Characteristics	Total (*N*)	Univariate analysis		Multivariate analysis	
		Hazard ratio (95% CI)^a^	*p* value	Hazard ratio (95% CI)	*p* value
Age	367				
≤65	163				
>65	204	1.620 (1.154–2.276)	0.005	2.293 (1.539–3.416)	<0.001
Gender	370				
Male	237				
Female	133	0.789 (0.554–1.123)	0.188	0.658 (0.441–0.982)	0.040
Histological type	369				
Papillary type	5				
Tubular type	69	0.559 (0.169–1.847)	0.340	0.085 (0.023–0.314)	<0.001
Mucinous type	19	0.169 (0.034–0.838)	0.030	0.032 (0.006–0.174)	<0.001
Signet ring type	11	1.425 (0.378–5.375)	0.601	0.158 (0.032–0.771)	0.023
Diffuse type	63	0.586 (0.177–1.947)	0.383	0.109 (0.028–0.425)	0.001
Not otherwise specified	202	0.691 (0.218–2.192)	0.530	0.112 (0.031–0.400)	<0.001
Histologic grade	361				
G1	10				
G2	134	1.648 (0.400–6.787)	0.489	3.010 (0.391–23.196)	0.290
G3	217	2.174 (0.535–8.832)	0.278	3.900 (0.506–30.061)	0.191
T stage	362				
T1 + T2	96				
T3	167	1.713 (1.103–2.660)	0.016	1.364 (0.795–2.341)	0.259
T4	99	1.729 (1.061–2.819)	0.028	1.437 (0.745–2.773)	0.279
N stage	352				
N0	107				
N1 + N2	171	1.640 (1.051–2.557)	0.029	1.362 (0.730–2.542)	0.331
N3	74	2.709 (1.669–4.396)	<0.001	2.199 (1.020–4.740)	0.044
M stage	352				
M0	327				
M1	25	2.254 (1.295–3.924)	0.004	2.002 (1.013–3.958)	0.046
Pathologic stage	347				
Stage I + II	160				
Stage III + IV	187	1.947 (1.358–2.793)	<0.001	1.092 (0.570–2.094)	0.790
DLAT	370	0.785 (0.577–1.069)	0.124	0.662 (0.464–0.944)	0.023

^a^ Hazard ratio (95% Cl): the first group as the reference.

### 3.5 Protein expression analysis

DLAT protein expression was analyzed using the TCGA‐STAD data on the UALCAN website. We found that DLAT protein expression was significantly upregulated in the GC group compared with the normal group. (Figure 3F). Next, the study analyzed normal gastric mucosa and GC tissues using the CAB003782 antibody from the HPA database to assess DLAT protein expression through immunohistochemical (IHC) staining pictures provided by HPA. The results indicated that the staining intensity of DLAT antibody in normal gastric mucosa tissue was weak, while the intensity of DLAT antibody in GC tissue was strong (Figure 3G). This suggested that DLAT protein was highly expressed in GC tissues compared to normal gastric mucosa. The expression of DLAT protein in GC tissues, as obtained from the HPA database, was consistent with the mRNA expression tested by TCGA‐STAD RNA‐seq data and our RT‐qPCR assay.

### 3.6 Reduction of gastric cancer cell viability after silencing DLAT expression in vitro

**FIGURE 4:**
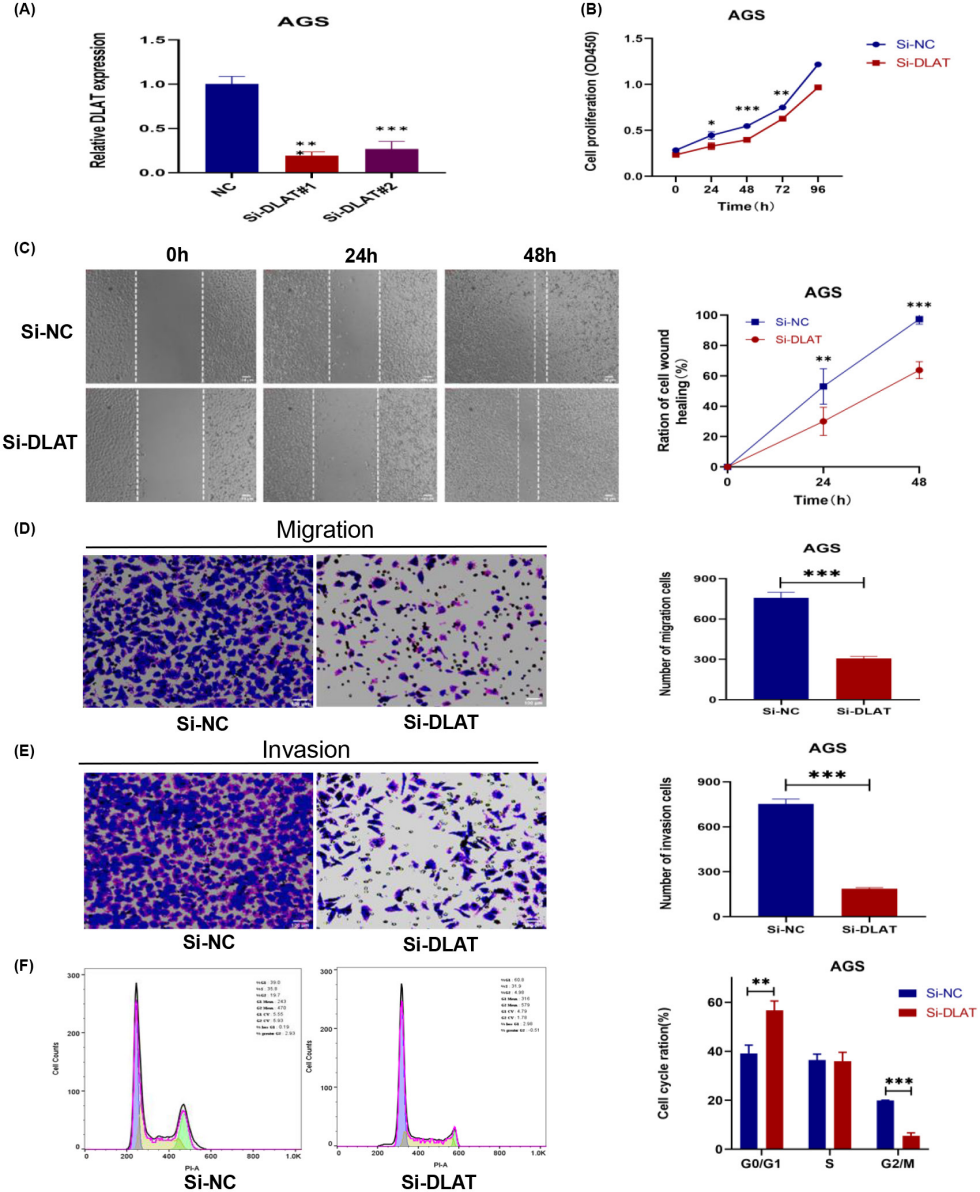
DLAT affected the proliferation, invasion and migration of gastric cancer cells (A). The siRNA silencing efficiency was detected by qRT‐PCR (B). CCK8 assay was performed to assess the proliferative ability of AGS cells after silencing DLAT (C). Wound healing assay to assess the migration of AGS cells after silencing DLAT (D). Transwell migration assay also to assess the migration ability of AGS cells after silencing DLAT (E). Transwell invasion assay to assess the invasion of AGS cells after silencing DLAT (F). Flow cytometry to assess the cycle of AGS cells after silencing DLAT. *p < 0.05, **p < 0.01, ***p < 0.001.

To silence the expression of DLAT, we transfected GC cells with si‐DLAT. Two si‐DLATs were designed. Firstly, we transfected AGS cells, which had the highest relative expression of DLAT among the cell lines, and detected the expression of DLAT after transfection of si‐DLAT using qRT‐PCR. We selected the siRNA with the highest silencing efficiency after 48 h of transfection for subsequent experiments (Figure 4A). The CCK8 assay was used to assess the proliferation ability of AGS cells. The results showed that the transfected Si‐DLAT group had a significantly reduced number of cell proliferation and weakened cell viability at 24, 48, and 72 h compared to the transfection‐treated Si‐NC group (Figure 4B). The result above suggested that DLAT might affect the proliferation ability of GC cells.

### 3.7 Silencing DLAT influences the migration of gastric cancer in vitro

To determine whether DLAT affected the migration ability of AGS cells, wound healing and transwell migration assays were performed. The wound healing assay displayed that compared to the control group transfected with Si‐NC, the migration of AGS cells at 24 h and 48 h after DLAT silencing was significantly reduced compared to the initial formation of scratches (0 h), the width of the scratches was markedly unnarrowed, and the ability of the scratches to heal was distinctly weakened (Figure 4C). A Transwell migration assay was performed to investigate the effect of DLAT on the migration ability of AGS cells. The results displayed that the number of AGS cells with silencing DLAT to the bottom chambers was significantly diminished compared to the Si‐NC group (Figure 4D). The above‐mentioned result revealed that the expression of DLAT affected the migration ability of AGS cells, which was significantly weakened after silencing DLAT.

### 3.8 DLAT probably influences the invasion of gastric cancer cells in vitro

We conducted a Transwell invasion assay to assess the impact of DLAT on the invasion ability of AGS cells. It was observed the number of cells transfected with Si‐DLAT that penetrated the gel into the lower chambers was greatly reduced compared to the transfected Si‐NC group (Figure 4E). This suggested that DLAT affected the invasion ability of AGS cells, and after silencing DLAT expression, the invasion ability of AGS cells was markedly weakened.

### 3.9 Silencing DLAT could arrest the G0/G1 stage and reduce the G2/M stage of gastric cancer in vitro

To evaluate the impact of DLAT on the GC cell cycle, we utilized flow cytometry to analyze the distribution and changes in the GC cell cycle after silencing DLAT. The findings indicated that the proportion of cell cycle in the G0/G1 phase was significantly increased and the proportion of the G2/M phase was decreased in the Si‐DLAT group compared to the Si‐NC group in AGS cells (Figure 4F). According to the above, silencing DLAT arrested the G0/G1 phase of the AGS cell cycle, and the proportion of the G2/M phase was reduced. These changes impacted the division and proliferation activity of the cells.

### 3.10 Analysis of protein–protein interactions and bioinformatics enrichment analysis of GO and KEGG pathways

**FIGURE 5:**
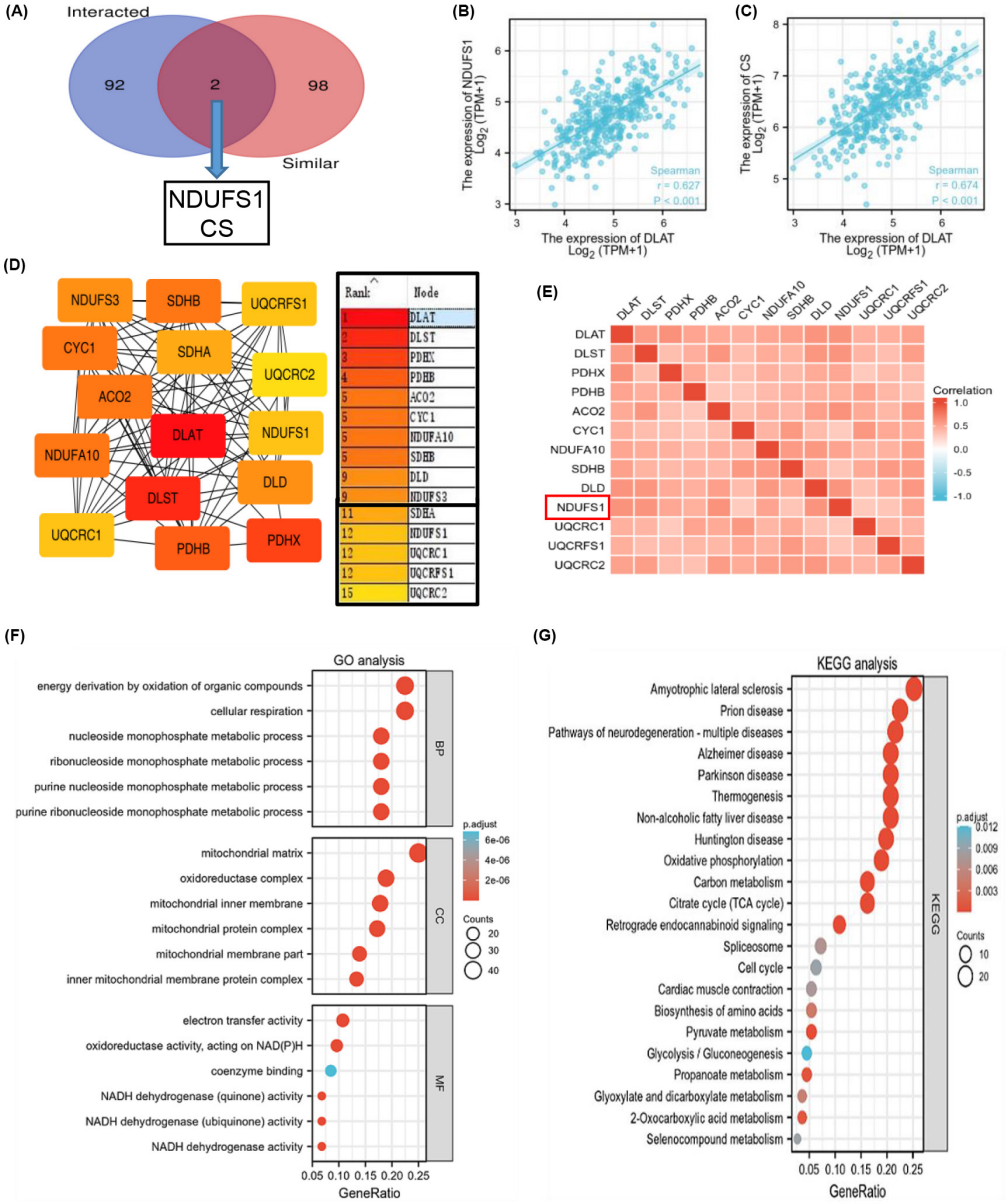
Proteins interaction analysis and GO and KEGG analyses (A). Intersection of gene sets from interaction proteins and similar genes of DLAT. Correlations in DLAT compared with NDUFS1 (B) and CS (C). (D) Top 15 proteins interacting most in two gene sets (E). Correlations of interaction proteins. GO (F) and KEGG (G) analyses. *p < 0.05, **p < 0.01, ***p < 0.001.

A Venn diagram was created using a set of genes that provide DLAT‐interacting proteins by STRING (experimental validation) and a set of genes providing functional similarity to DLAT in GC supplied by GEPIA2, and it was found that the intersection of the sets of genes was NDUFS1 and CS (Figure 5A). The analysis showed that there was a moderate positive correlation between the mRNA level expression of DLAT and NDUFS1 in GC tissues (*r* = 0.627, *p* < 0.001; Figure 5B), as well as a moderate positive correlation between the mRNA expression of DLAT and CS (*r* = 0.674, *p* < 0.001; Figure 5C). Cytoscape was used to analyze protein interactions for the gene sets mentioned above. As shown in Figure 5D, the top 15 nodal‐interacting proteins were listed by rank order, and NDUFS1 was found to be included. The correlation heatmap, created using TCGA‐STAD, analyzed the correlation between the above node genes and DLAT (Figure 5E). The results showed that NDUFS1 was also significantly correlated with DLAT. Subsequently, we performed GO analysis and KEGG enrichment analysis on the above gene sets to clarify the potential functions of DLAT protein, and drew a bubble diagram for visualization. The GO analysis yielded a total of 247 enriched biological processes (BP), 41 cytological components (CC), and 45 molecular biological functions (MF) under the requirements of *P*.adj < 0.05 and *q* value < 0.2, and the bubble diagrams were drawn for the top six of them (Figure 5F). BPs were mainly enriched in the oxidative energy acquisition of organic compounds and the process of cellular respiration; CCs were mainly enriched in the matrix part of mitochondria; MFs were mainly enriched in the electron transfer activity and the oxidoreductase activity acting on NAD(P)H. A total of 22 pathways were analyzed using KEGG enrichment under the conditions of P.adj <0.05 and *q* value < 0.2 (Figure 5G). Besides neurological specific diseases, the pathways were mainly enriched in oxidative phosphorylation, citric acid cycle, and retrograde endorphin signaling. The GO and KEGG pathway enrichment analyses indicated that DLAT was involved in the regulation of the biological process of cell cuproptosis.

### 3.11 Immune infiltration and immune checkpoint analysis

**FIGURE 6:**
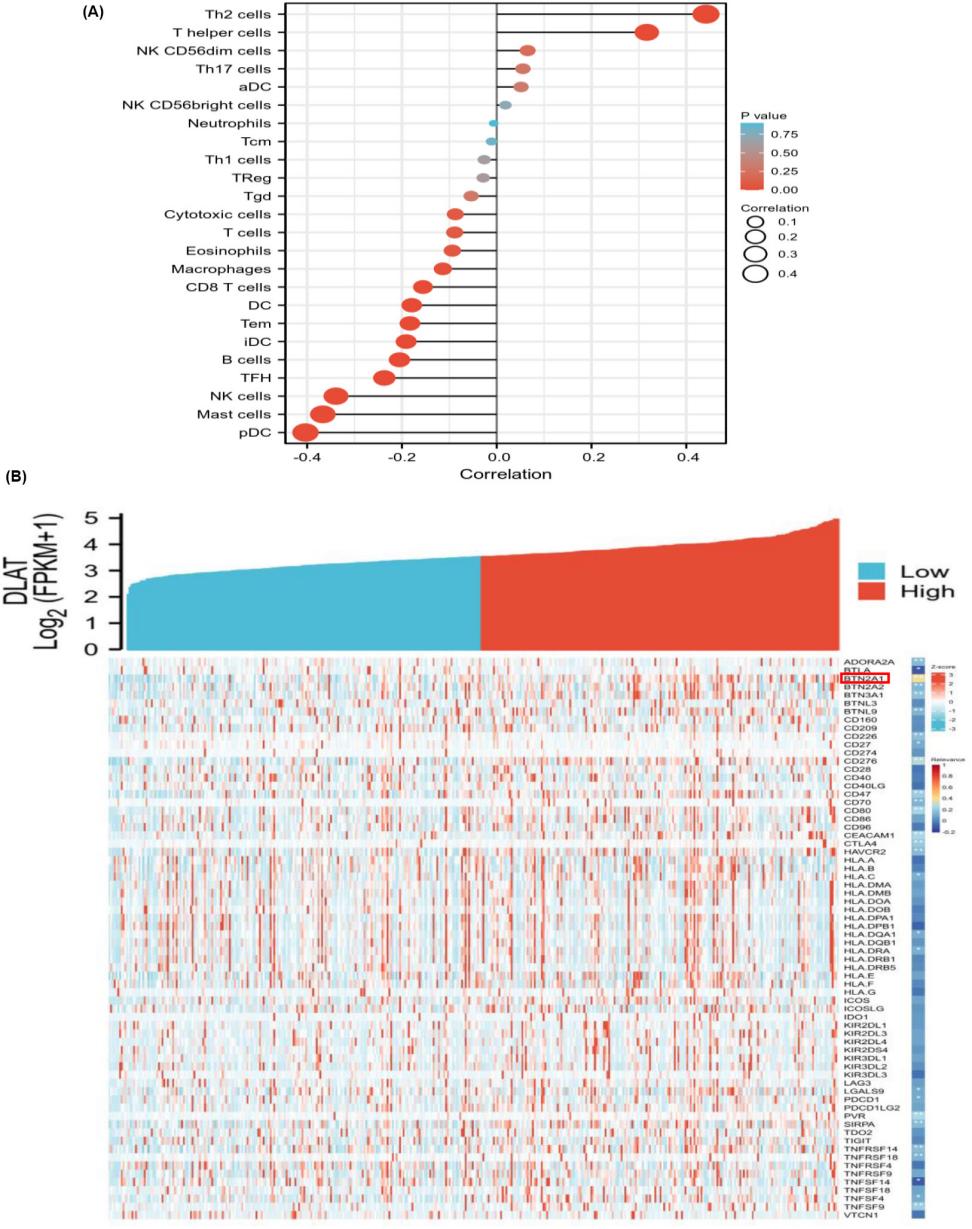
Immune infiltration analysis (A). Correlations between DLAT and Infiltrations of multiple immune cells in GC (B). Correlations between DLAT and different immune checkpoint genes. *p < 0.05, **p < 0.01, ***p < 0.001.

In this section, we assessed the correlation between DLAT expression and the degree of immune cell infiltration in GC. The findings showed that DLAT was significantly correlated with the degree of infiltration of several immune cells. DLAT was positively correlated with T helper type 2 (Th2) cells and T helper (Th) cells and negatively correlated with immune cells such as pDC (plasmacytoid DC) cells, mast cells, NK cells, TFH cells (T follicular helper cells), and B cells (Figure 6A). The DLAT expression from TCGA‐STAD was then ranked by median expression to perform co‐expression analysis with 79 immune checkpoint genes to analyze the molecular correlation. DLAT expression was positively correlated with BTN2A1 expression in GC (*p* < 0.01) and negatively correlated with the expression of 78 rest immune checkpoint genes (*p* < 0.05; Figure 6B). These results demonstrated that DLAT probably played an important role in immune cell infiltration and was involved in the regulation of tumor immune escape in GC.

## 4 DISCUSSION

As previously mentioned, GC is a complex and heterogeneous tumor with multiple risk factors, exhibiting epidemiological and histopathological differences among countries worldwide and showing tendencies for migration and recurrence.^37^ However, conventional treatments for GC have limited clinical efficacy, posing a significant challenge to researchers and clinical physicians. With the increasing understanding of GC pathogenesis and the identification of tumor‐resistant behaviors during targeted therapies, novel therapeutic options are emerging. These include additional biomarkers for diagnostic and prognostic purposes, targeted immune checkpoint inhibitors, and cell immunotherapies.^38^

Copper has been found to induce cell death through various mechanisms that differ from apoptosis, ferroptosis, cell autophagy, or programmed death. This suggests that cuproptosis may warrant further investigation as a potential avenue in tumor therapy. Previous research has proposed that copper carriers may be used to transport copper ions between cells of different tissues or to maintain intra‐ and extracellular copper homeostasis.^39^, ^40^ Additionally, they have been suggested as potential anticancer agents to induce copper overload in tumor cells.^41^ The process of cuproptosis, as elucidated by Peter et al.,^7^ involves the oligomerization of specific key proteins (e.g., DLAT) in mitochondria‐dependent TCA, resulting in cell death due to toxic protein stress after protein dysfunction. Therefore, it was important to comprehend the role of DLAT in inducing cellular cuproptosis in oxidative phosphorylation energy metabolism and its relevance in GC, where glycolysis serves as the primary way of energy metabolism. Consequently, the present study first aimed to investigate the involvement of DLAT, a gene associated with cuproptosis, in GC and its potential diagnostic and therapeutic implications. This study investigated the expression of DLAT in pan‐cancer and GC using bioinformatics and validated the upregulation of DLAT in GC using qRT‐PCR. Survival analysis revealed that GC patients with higher DLAT expression had a poorer prognosis, suggesting that DLAT may promote the progression of GC. These findings were consistent with previous observations linking high levels of DLAT expression to poor prognosis in pancreatic cancer,^42^ clear cell renal cell carcinoma,^43^ hepatocellular carcinoma,^8^, ^44^ and NSCLC.^45^ This indicated that DLAT has the potential to serve as a prognostic biomarker for the diagnosis and prognosis assessment in GC, in addition to our current findings. Furthermore, a series of cellular experiments were conducted to elucidate the specific effects of DLAT on the biological behavior of GC cells, revealing that DLAT expression not only affected cell proliferation, invasion, and migration but also affected the distribution of the cell cycle. Consistent with previous research by Lu et al., which demonstrated that OGDH‐mediated SIRT5, a metabolite of the TCA cycle, inhibited the proliferation and migration of GC cells,^46^ our findings support the notion that metabolite of the TCA cycle can impact the malignant biological behavior. Therefore, it is plausible that DLAT may function as an oncogene in driving GC progression. The findings have implications for guiding future studies on utilizing cuproptosis in precision treatment strategies for GC.

Currently, regulated cell death (RCD) processes such as apoptosis, autophagy, and ferroptosis have been implicated in tumorigenesis. These RCD processes are typically regulated by signaling molecules with specific biochemical, morphological, and immunological features.^47^ Peter et al.^7^ proposed that cuproptosis‐related genes, including DLAT, play a role in biological processes related to oxidative phosphorylation energy metabolism that regulate cellular copper metabolism. These genes are vital for mitochondrial aerobic respiration and the regulation of cell death induced by copper carriers. Metabolites of the TCA cycle SUCNR1 were found to be aberrantly expressed in GC and inhibited mitochondrial respiration.^48^ Our analysis of DLAT functional enrichment from GC RNA‐seq data suggested that its primary function was enriched in oxidative phosphorylation and the TCA cycle signaling pathways. Several studies have reported elevated copper levels in malignant tissues, including prostate cancer, lung cancer, glioblastoma and melanoma, breast cancer, and liver cancer.^49^ The dysregulation of copper metabolism is closely associated with tumourigenesis and progression.^50^ Copper as a transition metal in the signal transduction pathway, plays a crucial role in regulating the proliferation of cancer cells and tumor growth. Based on this, we hypothesized that copper overload disrupted the TCA cycle and subsequent mitochondrial respiration, which would promote the proliferation of GC cells and contribute to tumor growth. Given that solid tumors, including GC, often develop in low‐oxygen and low‐glucose environments due to inadequate vascular development, they primarily rely on glycolysis as their main metabolic pathway (Warburg effect) rather than oxidative phosphorylation. Glycolysis is a process whereby glucose is converted into lactic acid, which provides an efficient source of energy in glucose‐deficient conditions.^51^ It is well established that tumor cells predominantly utilize glycolysis over oxidative phosphorylation.^52^ Therefore, it would be beneficial to discuss the relationship between copper, DLAT, and cuproptosis. Unlike normal cells, which use oxidative phosphorylation in mitochondria for energy, GC cells also predominantly use the glycolytic pathway.^53^ However, other studies have also proposed that mitochondrial respiration and glycolysis pathways significantly influence the proliferation,^54^, ^55^ invasion,^56^, ^57^ and metabolic reprogramming^58^ behaviors of GC cells. DLAT, as part of the PDC complex, regulates the production of acetyl‐CoA linking glycolysis with the TCA cycle. This enzyme played a critical role in the metabolic reprogramming of cells,^59^ promoting rapid cell proliferation, invasion, and migration in tumor cells by enhancing energy production.^60^ In the context of GC cells, which are highly dependent on glycolysis, disruption of these pathways could indirectly lead to cell death. The cells were more dependent on mitochondrial respiration, copper ion overload can induce cuproptosis. Although the Warburg effect suggested a preference for glycolysis in GC cells, they were not completely incapable of using oxidative phosphorylation.^61^ Strategies to induce tumor cell death may include inhibition of their major metabolic pathways, and targeting glycolysis has been suggested to have significant antitumor effects. Research has shown that inhibiting key metabolic pathways, such as glycolysis, can induce tumor cell death.^16^ This could be achieved through the use of chemical inhibitors that block glycolytic processes within cells, potentially inhibiting tumor growth and progression.^62^ GC cells were primarily dependent on glycolysis, and interfering with their metabolic pathways could potentially indirectly lead to cell death. But how could this be used to induce tumor cell death? In addition, glycolysis was the process by which glucose was converted to lactic acid, which was an efficient source of energy under glucose‐deficient conditions.^51^ Impairing the conversion of pyruvate to lactate by reducing LDH‐A activity may enhance mitochondrial respiration. This not only impaired tumor cell proliferation under hypoxic conditions but may also increase their susceptibility to apoptosis by reducing the activation of BAX and BAD proteins. In our study, we found that the expression of DLAT was upregulated in GC cells and tissues, and the biological behaviors of GC cells, such as proliferation, invasion, and migration, were attenuated by silencing DLAT, reflecting its role as an oncogene. However, DLAT‐related cuproptosis requires the utilization of the oxidative phosphorylation pathway, whereas the Warburg effect in GC cells prevented the preferential utilization of the upregulated DLAT in GC cells. As mentioned above, GC cells were not completely incapable of using oxidative phosphorylation; it was just that glycolysis dominated their energy metabolism. Therefore, taking advantage of the upregulated expression of DLAT, supported by glycolysis inhibitors, and using copper carriers to induce cuproptosis in GC cells may become a potentially promising tumor therapeutic strategy. In addition, a multifaceted approach involving the improvement of mitochondrial function, regulation of metabolic homeostasis, substrate, and oxygen supply, inhibition of oxidative stress, and targeted pharmacological interventions could significantly enhance oxidative phosphorylation in GC cells and provide a potential avenue for effective treatment.

The tumor IME of GC is a complex interaction between cancer cells and immune cells that can either promote or inhibit tumor progression. The IME has become a popular area of research in tumor studies. The infiltration of various immune cells exhibited a high correlation with both tumor progression and prognosis. The high heterogeneity of the TME was an important feature of GC,^63^ and the infiltration of immune cells in the tumor was an important indicator for prognostic judgment and evaluation of therapeutic effectiveness in GC. Bian et al.^11^ found that the expression of the cuproptosis‐related gene CDKN2A was negatively correlated with macrophage infiltration in renal clear cell carcinoma. Feng et al.^64^ demonstrated a positive correlation between the cuproptosis‐related gene SERPINE1 and the infiltration level of NK cells and mast cells. Furthermore, plasma cells and B cells were negatively correlated with SERPINE1 in GC. Therefore, upregulation of SERPINE1 expression may result in the promotion of an inhibitory IME in GC. DLAT's metabolic role influenced the composition and activation of immune cells, including T cells, macrophages, and natural killer cells. Changes in metabolites such as lactate and ketones, influenced by DLAT, could modulate immune behavior, fostering either an immunosuppressive environment that benefited the cancer or stimulating an antitumor immune response. The changes in metabolite levels (such as lactate and ketones) could modulate immune cell behavior either promoting an immunosuppressive environment favorable to cancer or stimulating an immune response against the tumor. Consequently, it was imperative to investigate the relationship between DLAT and immune cells. As immunotherapy has emerged as a promising approach for cancer treatment, the role of T helper (Th) cells in antitumor immune responses has gained significant attention.^65^ The immunotherapeutic relevance of TH1 and TH2 subsets of Th cells, which were involved in molecular interactions with a variety of immune signaling pathways, have been investigated in cancer. Breast cancer patients with a poorer prognosis were found to have increased Th2 cytokines (IL‐4, IL‐10) and decreased Th1 cytokines (IFN‐γ, IL‐2, IL‐12).^66^ Th2 effector cells were dependent on IL‐4 production and stimulated the STAT6 signaling pathway to upregulate the GATA3 transcription factor.^67^, ^68^ The cytokines and chemokines secreted by Th2 cells have a tumor‐promoting effect.^69^ Boosting immunosuppressive Th2 and Treg cell activity promoted tumor immune responses and induced tumor growth.^70^ In our study, we investigated the correlation between DLAT expression and various normal immune cells within the TME of GC, providing critical insights into how metabolic pathways may influence immune responses in cancer. There was a complex interaction between metabolic enzymes, such as DLAT, and immune cell modulation.^71^ We found that the expression of DLAT in GC was positively correlated with T helper (Th) and Th2 cells. Th cells were critical in orchestrating immune responses.^72^ A positive correlation with DLAT indicated that as DLAT expression increases, so did the activity or presence of Th cells, potentially enhancing certain immune responses within the TME. Th2 cells have typically been associated with promoting humoral immunity and may contribute to an anti‐inflammatory environment.^73^ The association of Th2 cells with higher levels of DLAT may suggest that metabolic changes associated with DLAT may favor a Th2‐involved immune response. The expression of Th and Th2 cells was crucial for orchestrating adaptive immune responses and may promote a tumor‐favorable microenvironment. They could also promote tumor growth by affecting the TME. In addition, we found that DLAT expression was negatively correlated with plasmacytoid dendritic cells (pDCs), mast cells, natural killer cells (NK cells), TFH cells, and B cells. The pDCs are critical in antiviral responses and can stimulate antitumor immunity. A negative correlation between DLAT and other immune cells may indicate that increased DLAT expression may reduce the efficacy of antitumor immune responses. Mast cells are also associated with immunity and inflammation. Their negative correlation with DLAT could reflect a shift in the inflammatory state of the TME that either favors tumor growth or dampens effective immune surveillance. The decrease in NK cell activity or number associated with higher DLAT expression could facilitate tumor escape from immune surveillance. A negative correlation between TFH and DLAT could potentially affect the effectiveness of B‐cell responses within the TME. Essential for humoral immunity, the negative association with B cells may indicate that higher DLAT expression was associated with reduced antibody‐mediated immune responses, which could impact the control of tumor growth. We are reasonable to believe that targeting DLAT could potentially alter the immune environment by shifting the balance of immune cells toward more effective antitumor types. Thus, this suggested that increased DLAT expression might impair these cells' antitumor functions, facilitating immune evasion and tumor progression.

Metabolic reprogramming in tumor cells not only supported their proliferation but also reshaped the immune landscape and immune evasion mechanisms to favor tumor survival. Furthermore, tumor cells may activate the immune checkpoint pathway to prevent recognition by the immune system and inhibit the immune response.^74^, ^75^ Given that the tumor phenotype can be influenced by alterations to the components of the TME through the modulation of immune checkpoints,^76^ the search for new therapeutic strategies in GC will necessitate a comprehensive understanding of the tumor cells and the microenvironment. Despite the progress in tumor immunotherapy, most patients still failed to respond to or benefited from immune checkpoint blockade (ICB) therapy.^36^ Only a small proportion of patients demonstrated a response to this treatment.^77^ Therefore, biomarkers predicting patient response to ICB immunotherapy could be employed to evaluate the choice of therapeutic strategies and to identify potential targets for ICB treatment. BTN2A1 was an immune checkpoint gene that played a role in immune regulation.^78^ Additionally, it modulated immune evasion or activation in cancer.^79^ Cano et al.^80^ demonstrated that BTN2A1 was a target for Vγ9Vδ2 T cell cytotoxicity against malignant cells. This finding suggested that BTN2A1 may be a potential target for immunotherapy. In our study, correlation analysis with a large number of immune checkpoint genes revealed a moderate positive correlation only between DLAT and BTN2A1, suggesting DLAT has a potential relationship with BTN2A1 in the immune landscape of GC. This hypothesis suggested that DLAT may influence GC cells to participate in immune evasion by regulating the expression of the immune checkpoint gene BTN2A1. This indicated that when DLAT expression affected energy metabolism, the expression of BTN2A1 and DLAT1 was accompanied by a consistent change or functional alteration. This demonstrated a complex relationship in which altered energy metabolism influenced immune regulation. This interaction could modulate immune checkpoints, potentially altering the efficacy of immune checkpoint inhibitors used in cancer therapy. Moreover, an understanding of the impact of DLAT on BTN2A1 and other immune checkpoints may facilitate the development of novel combination therapies that target both metabolic pathways and immune checkpoints. Further studies could investigate the mechanisms by which DLAT expression affected different immune cells, with the potential to identify biomarkers for predicting treatment response or tailoring immunotherapies based on the metabolic and immune status of GC. The present study underscored the significance of investigating metabolic enzymes related to cuproptosis in the context of GC. It suggested that alterations in energy metabolism were not only essential for cancer cell survival but also profoundly influenced the immune landscape within the TME. Thus, changes in energy metabolic enzymes were not only integral to cancer progression but also to the shaping of immune responses in cancer. The impact of the mitochondria‐dependent energy metabolizing enzyme DLAT, which related cuproptosis, on the energy metabolism of tumor cells and its potential involvement as a therapeutic target in the immunotherapy of tumors remained to be elucidated. Additionally, it was demonstrated that intracellular copper levels could be controlled to selectively eliminate tumor cells.^19^ The next generation of selective copper carriers should use targeting sites that only be recognized by specific receptors. Additionally, these receptors should be present only in specific types of cancer cells, allowing the next generation of copper carriers to attain a more precise targeting effect. Our findings will also provide new insights into pharmacological therapeutic strategies for tumor treatments.

Immunonutrition was designed to enhance immune function and nutritional status in patients undergoing special treatments. Pusceddu et al.^81^ proposed a protocol to study the effects of immunonutrition supplementation on patients with resectable GC undergoing neoadjuvant chemotherapy with the FLOT (fluorouracil, leucovorin, oxaliplatin, and docetaxel) regimen. The study assessed the feasibility and safety of modifying dietary copper intake or adding copper supplements to the treatment regimen. Copper and immunonutrition were conducted in clinical trials to assess their combined impact on chemotherapy efficacy. This integrated strategy could improve the effectiveness of neoadjuvant chemotherapy in GC by increasing the susceptibility of cancer cells to treatment. However, the potential risks of copper supplementation, such as toxicity and the necessity of maintaining a delicate balance to avoid adverse effects, will also be critically evaluated. Regular monitoring of copper levels in patients will be crucial. Additionally, we could propose investigating DLAT and other cuproptosis‐related genes as biomarkers to predict responses to treatments modulated by copper. Ultimately, further research and clinical trials are essential to validate these concepts and refine treatment protocols for improved patient outcomes. Nevertheless, the precise role of induced tumor cell cuproptosis in cancer treatment remained unclear, and a large number of high‐quality basic studies would be required to prove the relationship between cuproptosis‐related genes and tumors.

## 5 CONCLUSION

This study analyzed the cuproptosis‐related gene DLAT expression profile and its prognostic risk assessment value in GC. DLAT may play an important role in the prognostic assessment of GC. The expression of DLAT could affect the biological behaviors of GC cell proliferation, invasion, and migration. Thus, taking advantage of the energy metabolism pathway of cuproptosis could hold promise for targeting tumor cell death through combination therapy. Additionally, it may be associated with tumor immune cell infiltration and involved in tumor immune escape by interfering with immune checkpoints. These findings suggested that cuproptosis‐related gene research may provide new treatment options for cancer.

## AUTHOR CONTRIBUTIONS

**Yanyu Peng:** Conceptualization (lead); data curation (supporting); formal analysis (supporting); funding acquisition (lead); methodology (equal); project administration (lead); resources (lead); writing – original draft (lead); writing – review and editing (supporting). **Ruimeng Shi:** Software (lead); validation (lead); visualization (supporting). **Siwen Yang:** Data curation (equal); formal analysis (equal); investigation (equal); supervision (equal); visualization (equal). **Jiayi Zhu:** Supervision (equal); writing – review and editing (equal).

## Supporting information


Data S1.

## Data Availability

The original data in the manuscript were downloaded from the official websites of TCGA (https://portal.gdc.cancer.gov/). The RNA‐seq data in TPM format were obtained from UCSC XENA (https://xenabrowser.net/datapages/).

## References

[1] Sung H , Ferlay J , Siegel RL , et al. Global cancer statistics 2020: GLOBOCAN estimates of incidence and mortality worldwide for 36 cancers in 185 countries. CA Cancer J Clin. 2021;71(3):209‐249. 33538338 10.3322/caac.21660

[2] Rocken C . Predictive biomarkers in gastric cancer. J Cancer Res Clin Oncol. 2023;149(1):467‐481. 36260159 10.1007/s00432-022-04408-0 PMC9889517

[3] Xia C , Basu P , Kramer BS , et al. Cancer screening in China: a steep road from evidence to implementation. Lancet Public Health. 2023;8(12):e996‐e1005. 38000379 10.1016/S2468-2667(23)00186-X PMC10665203

[4] Smyth EC , Nilsson M , Grabsch HI , van Grieken NC , Lordick F . Gastric cancer. Lancet. 2020;396(10251):635‐648. 32861308 10.1016/S0140-6736(20)31288-5

[5] Qiu H , Cao S , Xu R . Cancer incidence, mortality, and burden in China: a time‐trend analysis and comparison with the United States and United Kingdom based on the global epidemiological data released in 2020. Cancer Commun (Lond). 2021;41(10):1037‐1048. 34288593 10.1002/cac2.12197 PMC8504144

[6] Ruiz LM , Libedinsky A , Elorza AA . Role of copper on mitochondrial function and metabolism. Front Mol Biosci. 2021;8:711227. 34504870 10.3389/fmolb.2021.711227 PMC8421569

[7] Tsvetkov P , Coy S , Petrova B , et al. Copper induces cell death by targeting lipoylated TCA cycle proteins. Science. 2022;375(6586):1254‐1261. 35298263 10.1126/science.abf0529 PMC9273333

[8] Qin H , Sheng W , Zhang G , et al. Comprehensive analysis of cuproptosis‐related prognostic gene signature and tumor immune microenvironment in HCC. Front Genet. 2023;14:1094793. 36891150 10.3389/fgene.2023.1094793 PMC9986498

[9] Babak MV , Ahn D . Modulation of intracellular copper levels as the mechanism of action of anticancer copper complexes: clinical relevance. Biomedicine. 2021;9(8):852. 10.3390/biomedicines9080852 PMC8389626 34440056

[10] Gupta S , Roy A , Dwarakanath BS . Metabolic cooperation and competition in the tumor microenvironment: implications for therapy. Front Oncol. 2017;7:68. 28447025 10.3389/fonc.2017.00068 PMC5388702

[11] Bian Z , Fan R , Xie L . A novel Cuproptosis‐related prognostic gene signature and validation of differential expression in clear cell renal cell carcinoma. Genes (Basel). 2022;13(5):851. 35627236 10.3390/genes13050851 PMC9141858

[12] Chen D , Cui QC , Yang H , Dou QP . Disulfiram, a clinically used anti‐alcoholism drug and copper‐binding agent, induces apoptotic cell death in breast cancer cultures and xenografts via inhibition of the proteasome activity. Cancer Res. 2006;66(21):10425‐10433. 17079463 10.1158/0008-5472.CAN-06-2126

[13] Cen D , Brayton D , Shahandeh B , Meyskens FL Jr , Farmer PJ . Disulfiram facilitates intracellular Cu uptake and induces apoptosis in human melanoma cells. J Med Chem. 2004;47(27):6914‐6920. 15615540 10.1021/jm049568z

[14] Li J , Wu F , Li C , et al. The cuproptosis‐related signature predicts prognosis and indicates immune microenvironment in breast cancer. Front Genet. 2022;13:977322. 36226193 10.3389/fgene.2022.977322 PMC9548612

[15] Chang JH , Jiang Y , Pillarisetty VG . Role of immune cells in pancreatic cancer from bench to clinical application: an updated review. Medicine (Baltimore). 2016;95(49):e5541. 27930550 10.1097/MD.0000000000005541 PMC5266022

[16] Fantin VR , St‐Pierre J , Leder P . Attenuation of LDH‐A expression uncovers a link between glycolysis, mitochondrial physiology, and tumor maintenance. Cancer Cell. 2006;9(6):425‐434. 16766262 10.1016/j.ccr.2006.04.023

[17] Blockhuys S , Celauro E , Hildesjo C , et al. Defining the human copper proteome and analysis of its expression variation in cancers. Metallomics. 2017;9(2):112‐123. 27942658 10.1039/c6mt00202a

[18] Ishida S , Andreux P , Poitry‐Yamate C , Auwerx J , Hanahan D . Bioavailable copper modulates oxidative phosphorylation and growth of tumors. Proc Natl Acad Sci USA. 2013;110(48):19507‐19512. 24218578 10.1073/pnas.1318431110 PMC3845132

[19] Ge EJ , Bush AI , Casini A , et al. Connecting copper and cancer: from transition metal signalling to metalloplasia. Nat Rev Cancer. 2022;22(2):102‐113. 34764459 10.1038/s41568-021-00417-2 PMC8810673

[20] Erler JT , Bennewith KL , Cox TR , et al. Hypoxia‐induced lysyl oxidase is a critical mediator of bone marrow cell recruitment to form the premetastatic niche. Cancer Cell. 2009;15(1):35‐44. 19111879 10.1016/j.ccr.2008.11.012 PMC3050620

[21] Skrajnowska D , Bobrowska‐Korczak B , Tokarz A , Bialek S , Jezierska E , Makowska J . Copper and resveratrol attenuates serum catalase, glutathione peroxidase, and element values in rats with DMBA‐induced mammary carcinogenesis. Biol Trace Elem Res. 2013;156(1–3):271‐278. 24213724 10.1007/s12011-013-9854-x PMC3844146

[22] Yao K , Zhang R , Li L , et al. The signature of cuproptosis‐related immune genes predicts the tumor microenvironment and prognosis of prostate adenocarcinoma. Front Immunol. 2023;14:1181370. 37600770 10.3389/fimmu.2023.1181370 PMC10433769

[23] Vivian J , Rao AA , Nothaft FA , et al. Toil enables reproducible, open source, big biomedical data analyses. Nat Biotechnol. 2017;35(4):314‐316. 28398314 10.1038/nbt.3772 PMC5546205

[24] Bartha A , Gyorffy B . TNMplot.Com: a web tool for the comparison of gene expression in Normal, tumor and metastatic tissues. Int J Mol Sci. 2021;22(5):2622. 33807717 10.3390/ijms22052622 PMC7961455

[25] Love MI , Huber W , Anders S . Moderated estimation of fold change and dispersion for RNA‐seq data with DESeq2. Genome Biol. 2014;15(12):550. 25516281 10.1186/s13059-014-0550-8 PMC4302049

[26] Mangiola S , Doyle MA , Papenfuss AT . Interfacing Seurat with the R tidy universe. Bioinformatics. 2021;37:4100‐4107. 34028547 10.1093/bioinformatics/btab404 PMC9502154

[27] Gyorffy B . Survival analysis across the entire transcriptome identifies biomarkers with the highest prognostic power in breast cancer. Comput Struct Biotechnol J. 2021;19:4101‐4109. 34527184 10.1016/j.csbj.2021.07.014 PMC8339292

[28] Liu J , Lichtenberg T , Hoadley KA , et al. An integrated TCGA Pan‐cancer clinical data resource to drive high‐quality survival outcome analytics. Cell. 2018;173(2):400‐416 e411. 29625055 10.1016/j.cell.2018.02.052 PMC6066282

[29] Chandrashekar DS , Karthikeyan SK , Korla PK , et al. UALCAN: an update to the integrated cancer data analysis platform. Neoplasia. 2022;25:18‐27. 35078134 10.1016/j.neo.2022.01.001 PMC8788199

[30] Chandrashekar DS , Bashel B , Balasubramanya SAH , et al. UALCAN: a portal for facilitating tumor subgroup gene expression and survival analyses. Neoplasia. 2017;19(8):649‐658. 28732212 10.1016/j.neo.2017.05.002 PMC5516091

[31] Uhlen M , Fagerberg L , Hallstrom BM , et al. Proteomics. Tissue‐based map of the human proteome. Science. 2015;347(6220):1260419. 25613900 10.1126/science.1260419

[32] Szklarczyk D , Gable AL , Nastou KC , et al. Correction to ‘The STRING database in 2021: customizable protein‐protein networks, and functional characterization of user‐uploaded gene/measurement sets’. Nucleic Acids Res. 2021;49(18):10800. 34530444 10.1093/nar/gkab835 PMC8501959

[33] Otasek D , Morris JH , Boucas J , Pico AR , Demchak B . Cytoscape automation: empowering workflow‐based network analysis. Genome Biol. 2019;20(1):185. 31477170 10.1186/s13059-019-1758-4 PMC6717989

[34] Hanzelmann S , Castelo R , Guinney J . GSVA: gene set variation analysis for microarray and RNA‐seq data. BMC Bioinformatics. 2013;14:7. 23323831 10.1186/1471-2105-14-7 PMC3618321

[35] Bindea G , Mlecnik B , Tosolini M , et al. Spatiotemporal dynamics of intratumoral immune cells reveal the immune landscape in human cancer. Immunity. 2013;39(4):782‐795. 24138885 10.1016/j.immuni.2013.10.003

[36] Hu FF , Liu CJ , Liu LL , Zhang Q , Guo AY . Expression profile of immune checkpoint genes and their roles in predicting immunotherapy response. Brief Bioinform. 2021;22(3):bbaa176. 32814346 10.1093/bib/bbaa176

[37] Chia NY , Tan P . Molecular classification of gastric cancer. Ann Oncol. 2016;27(5):763‐769. 26861606 10.1093/annonc/mdw040

[38] Yang WJ , Zhao HP , Yu Y , et al. Updates on global epidemiology, risk and prognostic factors of gastric cancer. World J Gastroenterol. 2023;29(16):2452‐2468. 37179585 10.3748/wjg.v29.i16.2452 PMC10167900

[39] Gioilli BD , Kidane TZ , Fieten H , et al. Secretion and uptake of copper via a small copper carrier in blood fluid. Metallomics. 2022;14(3):mfac006. 35199838 10.1093/mtomcs/mfac006 PMC8962702

[40] Hatori Y , Lutsenko S . The role of copper chaperone Atox1 in coupling redox homeostasis to intracellular copper distribution. Antioxidants (Basel). 2016;5(3):25. 27472369 10.3390/antiox5030025 PMC5039574

[41] Zhang J , Duan D , Xu J , Fang J . Redox‐dependent copper carrier promotes cellular copper uptake and oxidative stress‐mediated apoptosis of cancer cells. ACS Appl Mater Interfaces. 2018;10(39):33010‐33021. 30209950 10.1021/acsami.8b11061

[42] Fang Z , Wang W , Liu Y , et al. Cuproptosis‐related gene DLAT as a novel biomarker correlated with prognosis, Chemoresistance, and immune infiltration in pancreatic adenocarcinoma: a preliminary study based on bioinformatics analysis. Curr Oncol. 2023;30(3):2997‐3019. 36975441 10.3390/curroncol30030228 PMC10047569

[43] Huang S , Cai C , Zhou K , et al. Cuproptosis‐related gene DLAT serves as a prognostic biomarker for immunotherapy in clear cell renal cell carcinoma: multi‐database and experimental verification. Aging (Albany NY). 2023;15(21):12314‐12329. 37938155 10.18632/aging.205181 PMC10683628

[44] Zhang P , Zhao JH , Yuan LX , et al. DLAT is a promising prognostic marker and therapeutic target for hepatocellular carcinoma: a comprehensive study based on public databases. Sci Rep. 2023;13(1):17295. 37828099 10.1038/s41598-023-43835-y PMC10570290

[45] Chen Q , Wang Y , Yang L , et al. PM2.5 promotes NSCLC carcinogenesis through translationally and transcriptionally activating DLAT‐mediated glycolysis reprograming. J Exp Clin Cancer Res. 2022;41(1):229. 35869499 10.1186/s13046-022-02437-8 PMC9308224

[46] Lu X , Yang P , Zhao X , et al. OGDH mediates the inhibition of SIRT5 on cell proliferation and migration of gastric cancer. Exp Cell Res. 2019;382(2):111483. 31247190 10.1016/j.yexcr.2019.06.028

[47] Tang D , Kang R , Berghe TV , Vandenabeele P , Kroemer G . The molecular machinery of regulated cell death. Cell Res. 2019;29(5):347‐364. 30948788 10.1038/s41422-019-0164-5 PMC6796845

[48] Rabe P , Liebing AD , Krumbholz P , Kraft R , Staubert C . Succinate receptor 1 inhibits mitochondrial respiration in cancer cells addicted to glutamine. Cancer Lett. 2022;526:91‐102. 34813893 10.1016/j.canlet.2021.11.024

[49] Chakravarty R , Chakraborty S , Dash A . (64)Cu(2+) ions as PET probe: an emerging paradigm in molecular imaging of cancer. Mol Pharm. 2016;13(11):3601‐3612. 27709959 10.1021/acs.molpharmaceut.6b00582

[50] Zhao S , Zhang X , Gao F , et al. Identification of copper metabolism‐related subtypes and establishment of the prognostic model in ovarian cancer. Front Endocrinol (Lausanne). 2023;14:1145797. 36950684 10.3389/fendo.2023.1145797 PMC10025496

[51] Niu D , Luo T , Wang H , Xia Y , Xie Z . Lactic acid in tumor invasion. Clin Chim Acta. 2021;522:61‐69. 34400170 10.1016/j.cca.2021.08.011

[52] El Imad T , El Khoury L , Geara AS . Warburg's effect on solid tumors. Saudi J Kidney Dis Transpl. 2014;25(6):1270‐1277. 25394449 10.4103/1319-2442.144266

[53] Wu T , Yang Y , Zhang B , et al. EDDM3A drives gastric cancer progression by promoting HIF‐1alpha‐dependent aerobic glycolysis. Oncogene. 2022;11(1):3. 10.1038/s41389-022-00379-6 PMC8764035 35039478

[54] Shen Y , Yang J , Li J , et al. Carnosine inhibits the proliferation of human gastric cancer SGC‐7901 cells through both of the mitochondrial respiration and glycolysis pathways. PLoS One. 2014;9(8):e104632. 25115854 10.1371/journal.pone.0104632 PMC4130552

[55] Zhu H , Chan KT , Huang X , et al. Cystathionine‐beta‐synthase is essential for AKT‐induced senescence and suppresses the development of gastric cancers with PI3K/AKT activation. elife. 2022;11:11. 10.7554/eLife.71929 PMC9236611 35758651

[56] Shida M , Kitajima Y , Nakamura J , et al. Impaired mitophagy activates mtROS/HIF‐1alpha interplay and increases cancer aggressiveness in gastric cancer cells under hypoxia. Int J Oncol. 2016;48(4):1379‐1390. 26820502 10.3892/ijo.2016.3359

[57] Rho M , Kim J , Jee CD , et al. Expression of type 2 hexokinase and mitochondria‐related genes in gastric carcinoma tissues and cell lines. Anticancer Res. 2007;27(1A):251‐258. 17352240

[58] Chang TC , Lee HT , Pan SC , et al. Metabolic reprogramming in response to alterations of mitochondrial DNA and mitochondrial dysfunction in gastric adenocarcinoma. Int J Mol Sci. 2022;23(3):1857. 35163779 10.3390/ijms23031857 PMC8836428

[59] Matsuda S , Adachi J , Ihara M , et al. Nuclear pyruvate kinase M2 complex serves as a transcriptional coactivator of arylhydrocarbon receptor. Nucleic Acids Res. 2016;44(2):636‐647. 26405201 10.1093/nar/gkv967 PMC4737187

[60] Sun XR , Sun Z , Zhu Z , et al. Expression of pyruvate dehydrogenase is an independent prognostic marker in gastric cancer. World J Gastroenterol. 2015;21(17):5336‐5344. 25954108 10.3748/wjg.v21.i17.5336 PMC4419075

[61] Liu YD , Yu L , Ying L , et al. Toll‐like receptor 2 regulates metabolic reprogramming in gastric cancer via superoxide dismutase 2. Int J Cancer. 2019;144(12):3056‐3069. 30536754 10.1002/ijc.32060 PMC6590666

[62] Vennin C , Cattaneo CM , Bosch L , et al. Taxanes trigger cancer cell killing in vivo by inducing non‐canonical T cell cytotoxicity. Cancer Cell. 2023;41(6):1170‐1185.e1112. 37311414 10.1016/j.ccell.2023.05.009

[63] Chen J , Liu K , Luo Y , et al. Single‐cell profiling of tumor immune microenvironment reveals immune irresponsiveness in gastric signet‐ring cell carcinoma. Gastroenterology. 2023;165(1):88‐103. 36921674 10.1053/j.gastro.2023.03.008

[64] Feng L , Li G , Li D , Duan G , Liu J . Cuproptosis‐related gene SERPINE1 is a prognostic biomarker and correlated with immune infiltrates in gastric cancer. J Cancer Res Clin Oncol. 2023;149(12):10851‐10865. 37318594 10.1007/s00432-023-04900-1 PMC10423162

[65] Borst J , Ahrends T , Babala N , Melief CJM , Kastenmuller W . CD4(+) T cell help in cancer immunology and immunotherapy. Nat Rev Immunol. 2018;18(10):635‐647. 30057419 10.1038/s41577-018-0044-0

[66] Hong CC , Yao S , McCann SE , et al. Pretreatment levels of circulating Th1 and Th2 cytokines, and their ratios, are associated with ER‐negative and triple negative breast cancers. Breast Cancer Res Treat. 2013;139(2):477‐488. 23624818 10.1007/s10549-013-2549-3 PMC3912696

[67] Saravia J , Chapman NM , Chi H . Helper T cell differentiation. Cell Mol Immunol. 2019;16(7):634‐643. 30867582 10.1038/s41423-019-0220-6 PMC6804569

[68] Luckheeram RV , Zhou R , Verma AD , Xia B . CD4(+)T cells: differentiation and functions. Clin Dev Immunol. 2012;2012:925135. 22474485 10.1155/2012/925135 PMC3312336

[69] Basu A , Ramamoorthi G , Albert G , et al. Differentiation and regulation of T(H) cells: a balancing act for cancer immunotherapy. Front Immunol. 2021;12:669474. 34012451 10.3389/fimmu.2021.669474 PMC8126720

[70] Fu C , Jiang L , Hao S , et al. Activation of the IL‐4/STAT6 signaling pathway promotes lung cancer progression by increasing M2 myeloid cells. Front Immunol. 2019;10:2638. 31798581 10.3389/fimmu.2019.02638 PMC6863933

[71] Peng‐Winkler Y , Wessels I , Rink L , Fischer HJ . Zinc levels affect the metabolic switch of T cells by modulating glucose uptake and insulin receptor signaling. Mol Nutr Food Res. 2022;66(9):e2100944. 35182109 10.1002/mnfr.202100944

[72] Nakamura A , Haroon N . Recent updates in the immunopathology of type 3 immunity‐mediated enthesitis. Curr Rheumatol Rep. 2021;23(5):31. 33893896 10.1007/s11926-021-00995-y

[73] Jay DC , Nadeau KC . Immune mechanisms of sublingual immunotherapy. Curr Allergy Asthma Rep. 2014;14(11):473. 25195100 10.1007/s11882-014-0473-1

[74] Beatty GL , Gladney WL . Immune escape mechanisms as a guide for cancer immunotherapy. Clin Cancer Res. 2015;21(4):687‐692. 25501578 10.1158/1078-0432.CCR-14-1860 PMC4334715

[75] Marin‐Acevedo JA , Dholaria B , Soyano AE , Knutson KL , Chumsri S , Lou Y . Next generation of immune checkpoint therapy in cancer: new developments and challenges. J Hematol Oncol. 2018;11(1):39. 29544515 10.1186/s13045-018-0582-8 PMC5856308

[76] Li Y , Hu X , Lin R , et al. Single‐cell landscape reveals active cell subtypes and their interaction in the tumor microenvironment of gastric cancer. Theranostics. 2022;12(8):3818‐3833. 35664061 10.7150/thno.71833 PMC9131288

[77] Sharma P , Hu‐Lieskovan S , Wargo JA , Ribas A . Primary, adaptive, and acquired resistance to cancer immunotherapy. Cell. 2017;168(4):707‐723. 28187290 10.1016/j.cell.2017.01.017 PMC5391692

[78] Incorvaia L , Fanale D , Badalamenti G , et al. Baseline plasma levels of soluble PD‐1, PD‐L1, and BTN3A1 predict response to nivolumab treatment in patients with metastatic renal cell carcinoma: a step toward a biomarker for therapeutic decisions. Onco Targets Ther. 2020;9(1):1832348. 10.1080/2162402X.2020.1832348 PMC7595592 33178494

[79] Incorvaia L , Rinaldi G , Badalamenti G , et al. Prognostic role of soluble PD‐1 and BTN2A1 in overweight melanoma patients treated with nivolumab or pembrolizumab: finding the missing links in the symbiotic immune‐metabolic interplay. Ther Adv Med Oncol. 2023;15:17588359231151845. 36818688 10.1177/17588359231151845 PMC9936535

[80] Cano CE , Pasero C , De Gassart A , et al. BTN2A1, an immune checkpoint targeting Vgamma9Vdelta2 T cell cytotoxicity against malignant cells. Cell Rep. 2021;36(2):109359. 34260935 10.1016/j.celrep.2021.109359

[81] Pusceddu V , Donisi C , Pretta A , et al. Immunonutrition supplementation for resectable gastric cancer during standard neoadjuvant chemotherapy of FLOT. A proof‐of‐concept protocol: I‐SUPPLY. ESMO Gastrointestinal Oncology. 2024;3:100036. 10.1016/j.esmogo.2023.100036 PMC12836635 41648748

