# Supplementary material for: Cuproptosis‐related gene DLAT is a biomarker of the prognosis and immune microenvironment of gastric cancer and affects the invasion and migration of cells
Source: Cancer Med. 2024 Jul 19;13(14):e70012. doi: 10.1002/cam4.70012 (PMC11258438; doi:10.1002/cam4.70012)
Supplement: Supplementary file 1 — Data S1. [file CAM4-13-e70012-s001.docx]

Supplemental Abbreviations Table

**Abbreviations**

| Abbreviation | Full name |
| --- | --- |
| ACC | adrenocortical carcinoma |
| BLCA | bladder urothelial carcinoma |
| BRCA | breast invasive carcinoma |
| CESC | cervical squamous cell carcinoma and endocervical adenocarcinoma |
| CHOL | cholangiocarcinoma |
| COAD | colon adenocarcinoma |
| DLBC | lymphoid neoplasm diffuse large b-cell lymphoma |
| ESCA | esophageal carcinoma |
| GBM | glioblastoma multiforme |
| HNSC | head and neck squamous carcinoma |
| KICH | kidney chromophobe |
| KIRC | kidney renal clear cell carcinoma |
| KIRP | kidney renal papillary cell carcinoma |
| LAML | acute myeloid leukemia |
| LGG | lower grade glioma |
| LIHC | liver cancer |
| LUAD | lung adenocarcinoma |
| LUSC | lung squamous cell carcinoma |
| MESO | mesothelioma |
| OV | ovarian cancer |
| PAAD | pancreatic cancer |
| PCPG | pheochromocytoma & paraganglioma |
| PRAD | prostate cancer |
| READ | rectal cancer |
| SARC | sarcoma |
| SKCM | melanoma |
| STAD | stomach cancer |
| TGCT | testicular cancer |
| THCA | thyroid cancer |
| THYM | thymoma |
| UCEC | endometrioid cancer |
| UCS | uterine carcinosarcoma |
| UVM | ocular melanomas |
